# The Global Decline in Human Fertility: The Post-Transition Trap Hypothesis

**DOI:** 10.3390/life14030369

**Published:** 2024-03-11

**Authors:** Robert John Aitken

**Affiliations:** 1 Priority Research Centre for Reproductive Science, Discipline of Biological Sciences, School of Environmental and Life Sciences, College of Engineering Science and Environment, University of Newcastle, Callaghan, NSW 2308, Australia; john.aitken@newcastle.edu.au; Tel.: +61-2-4921-6851; 2 Hunter Medical Research Institute, New Lambton Heights, NSW 2305, Australia

**Keywords:** human population, first demographic transition, second demographic transition, total fertility rate, fecundity, socioeconomic factors, evolutionary factors, relaxed selection hypothesis, population decline

## Abstract

Over the past half a century many countries have witnessed a rapid fall in total fertility rates, particularly in the world’s most advanced economies including the industrial powerhouses of Eastern Asia and Europe. Such nations have now passed through the first and second demographic transitions and are currently exhibiting fertility rates well below the replacement threshold of 2.1, with no sign of recovery. This paper examines the factors responsible for driving these demographic transitions and considers their impact on both fertility and fecundity (our fundamental capacity to reproduce). I argue that because the first demographic transition was extremely rapid and largely driven by socioeconomic factors, it has had no lasting impact on the genetic/epigenetic underpinnings of human fecundity. However, the second demographic transition will be different. A series of conditions associated with low fertility societies, including relaxed selection pressure for high-fertility genotypes, the indiscriminate use of assisted reproductive technologies to treat human infertility, and environmental contamination with reproductive toxicants, may impact our genetic constitution in ways that compromise the future fecundity of our species. Since any fundamental change in the genetic foundations of human reproduction will be difficult to reverse, we should actively pursue methods to monitor human fecundity, as sub-replacement fertility levels become established across the globe.

## 1. Introduction

In the mid 1960’s, the rate of world population growth suddenly declined in concert with the widespread onset of a demographic transition [1,2,3]. This recent change appears to be near universal and instigated by the global increase in prosperity that followed the post-war expansion of world trade, as well as changes in education, healthcare and the *raisons d’être* that accompany socioeconomic development [4]. As a consequence of these trends, many of the most prosperous nations on Earth now exhibit total fertility rates (TFRs, i.e., the estimated number of children born to a woman during her reproductive lifespan) that are well below the replacement threshold of 2.1 children per woman. This trend has been particularly prominent in the tiger economies of Asia (South Korea, Taiwan, Singapore and Hong Kong) that now exhibit some of the lowest fertility rates in the world [2]; however, other nations are on the same demographic path. Indeed, Vollset et al. [5] predict that by 2050, this demographic convergence will result in 151 of 195 countries having a TFR lower than the replacement level, while 183 are predicted to pass this threshold by 2100. Given the inherent complexity of population dynamics in different countries, such predictions are extremely difficult to validate and have been mired in controversy [6,7,8]. Significant improvements in our capacity to predict future population trends will only be achieved once we have a better understanding of the factors responsible for the decline in fertility that accompanies the demographic transition. As a result, the latter has been the subject of extensive research [9,10,11,12,13].

I approach this topic from the perspective of a reproductive biologist with an interest in the fundamental mechanisms underpinning population change. In searching for factors responsible for low fertility rates across the globe and predicting how these trends might evolve in the future, I shall only briefly reference the cultural, social and economic differences that have both nuanced and dominated the literature in the past. Of course, every country is on its own individual journey, but the ultimate destination appears to be shared. In order to understand the dramatic demographic changes that we have witnessed over the last half century, we need to broaden our theoretical frameworks to encompass not just a wide range of socioeconomic states and a diversity of cultural backgrounds [14] but also the myriad biological and clinical determinants of reproductive health. In the microcosm of this discussion on demographic mechanisms, the elements that unite us are much more interesting and important than the differences that divide.

The aims of this review are: (i) to describe the demographic changes that are occurring to human populations across the globe; (ii) to review the fundamental mechanisms involved in precipitating the observed decline in fertility rates, from the powerful socioeconomic factors that are limiting family size in the short-term to the long-term genetic/epigenetic changes that may leave a more permanent mark on our fundamental capacity to procreate; and (iii) to hypothesize where these changes might lead us if concerted action is not taken.

## 2. Defining Terms—Measures of Fertility

Before initiating this discussion, it is important that we define terms. In particular, the importance of discriminating between fertility and fecundity is worth emphasizing because this distinction has a major bearing on our capacity to understand and predict human population trends. In biological terms, fecundity refers to the probability of achieving conception within any given menstrual/oestrus cycle. It is a basic biological concept that is powerfully impacted by long-term genetic and environmental factors and defines our fundamental capacity to reproduce [15]. The total fertility rate, on the other hand, is an output measure that refers to the average number of children a women would have in her reproductive lifetime, assuming she experiences current age-specific fertility rates. This distinction between fertility and fecundity is not consistently understood in the literature; indeed, these terms are often used interchangeably without reference to their precise biological meaning [16,17].

As a measure of fertility, TFR has the advantage of being a widely understood and accepted standardized measure that can be readily used for making comparisons between groups. However, it is a theoretical projection that does not necessarily indicate the actual number of children that a given women will give birth to in her lifetime. In particular, TFR is sensitive to shifts in the timing of childbirth [18] and may underestimate completed fertility when births are being postponed. To achieve an accurate assessment of a fertility rate, we would have to wait until women had come to the end of their reproductive life and then retrospectively calculate the ‘cohort completed fertility (CCF)’ or ‘completed fertility rate (CFR)’. The disadvantage of this approach is that it can only be applied to women born at least 49 years ago and cannot give an assessment of contemporary fertility levels. The advantage is that CCF gives the actual childbearing experience of real cohorts of women, not a projected value, and is untrammelled by fluctuations in reproductive behaviour. Nevertheless, these fertility measures are closely related [19,20] and both methodologies indicate that fertility rates are in global decline [21,22]. Throughout this manuscript, I have focused on the use of TFR as a fertility measure; however, the assumptions underpinning this term should always be borne in mind.

## 3. Pre-Transition Phase of Human Evolution

In pre-transition societies, fertility and fecundity are intimately related. Our species is characterized by the generation of immature, neotenous offspring that require many years of nurturing to reach a level of maturity commensurate with autonomy. Given the relatively short lifespan of our species during the earliest stages of its evolution, a quality–quantity trade off developed that limited our fecundity to 0.2–0.3, i.e., a 20–30% chance of becoming pregnant in a given menstrual cycle [23,24,25]. This fecundity setting may be low compared many animal species [26,27]; however, it was sufficient to allow neolithic women to produce an average of 4–6 children during their reproductive lifespan [28,29,30,31]. Of course, this figure is far below the theoretical maximum for our species as exemplified by the Hutterites who, freed from the constraints imposed by lactational amenorrhea, contraception and disease managed to generate average family sizes of ~10 [32]. However, the Hutterite example is not typical of pre-transition societies where lactational amenorrhoea would have been the major mechanism of birth spacing, contraception was not available, and disease would have been an ever-present threat. An average family size of 4–6 also resonates with data on present-day hunter gatherers for whom the average is 4.7 [33]. Since at least half of these children would never reach sexual maturity and have children of their own [34], birth rates and death rates were more or less balanced and allowed human population numbers to remain relatively stable throughout much of history.

In such pre-transition societies, population growth was largely limited by the Malthusian trap [35]. If resources, particularly food, were abundant, then the population would expand to the point that the resource became exhausted, leading to starvation, increased mortality and a corresponding decline in population numbers [11]. The Malthusian principle, coupled with the ravages of disease and war, were the chains that held our population in check since the dawn of humankind—but then, 250 years ago, everything changed.

## 4. Fertility and the Demographic Transition

The unprecedented growth of the human population has its origins in 18th century Europe and the initiation of Stage 2 of the demographic transition [36]. At that time, birth rates were still high, but death rates started to decline as a consequence of the increases in primary healthcare, security, prosperity and urbanization associated with the industrial revolution. The latter drove the generation of factories and the movement of labour away from the land to the cities. Such changes, coupled with the availability of international trade routes, the ready availability of coal, stable political systems, the emergence of capitalism and the development of novel means of transport, particularly the locomotive, fuelled the socioeconomic transformation of Britain and Northern Europe in the 18th and 19th centuries [37]. This sudden wave of prosperity was associated with a population explosion that might have resulted in a disaster of Malthusian proportions, had it not been for a parallel transformation in the efficiency of food production. This agricultural revolution not only enabled the rural work force to migrate to the cities in support of the industrial revolution but also allowed the population to escape the Malthusian trap [38]. As a result, the UK population expanded from 5 million in 1700 to over 9 million by 1801 and over 30 million by the end of the 19th century.

A global succession of technological and industrial revolutions subsequently created a wave of economic prosperity that has swept the world, first in mainland Europe, then the US, Asia and South America. Even sub-Saharan Africa, which still contains some of the poorest nations on Earth, is now showing the green shoots of economic prosperity—and, whenever such economic transformation occurs, it is accompanied by a demographic transition. The key to initiating the transition generally involves an increase in socioeconomic development that provides the resources, security and knowledge necessary to reduce infant/childhood mortality rates. Populations respond to such developments with a transient increase in fertility rate; however, as prosperity continues to grow, this brief phase of population growth is followed by a very non-Malthusian, reduction in fertility that comprises Stage 3 of the demographic transition. During this paradoxical process, fertility rates decline, while mortality rates are low and the resources needed to sustain the population are increasing.

**Figure 1:**
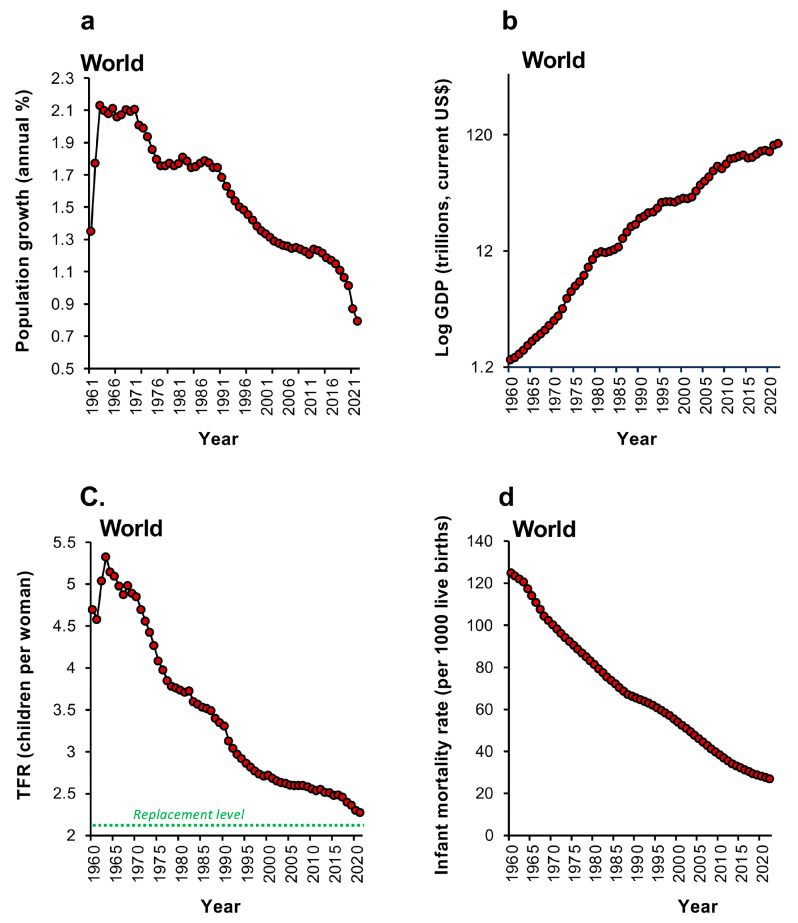
The global demographic transition since 1960. (**a**) The rate of global population growth suddenly tailed off in 1964 and then began its downward descent in 1972, accelerating over the past decade. (**b**) The increase in global prosperity over the past 62 years as reflected in log GDP in current $US. (**c**) The total fertility rate (TFR) initially increased but, beginning in 1963, began its inexorable descent towards the replacement threshold of 2.1 children per woman. (**d**) Over the same period of time, a progressive decrease in infant mortality has been evident and was a major factor in triggering the demographic transition. Each red circle represents data for a single year. Source: United Nations Department of Economic and Social Affairs, Population Division (2022).

By accessing databases such as the World Bank Open Data resource [21] and United Nations World Population Prospects [3], we can see exactly this chain of events played out across the globe. Thus, the aggregated world data presented in Figure 1 show that, as world prosperity grew from 1960 onwards, so the rate of population growth plateaued in 1964 before commencing a progressive decline in 1971 (Figure 1a,b). Between 1961 and 1963, the global TFR exhibited a transient increase (Figure 1c), largely due to the fertility recovery observed in China after the famine and social upheavals during the “great leap forward” period [39]. This trend then dramatically reversed in a steady descent towards replacement level fertility over the ensuing half century, in parallel with a global decline in infant mortality rates (Figure 1d).

**Figure 2:**
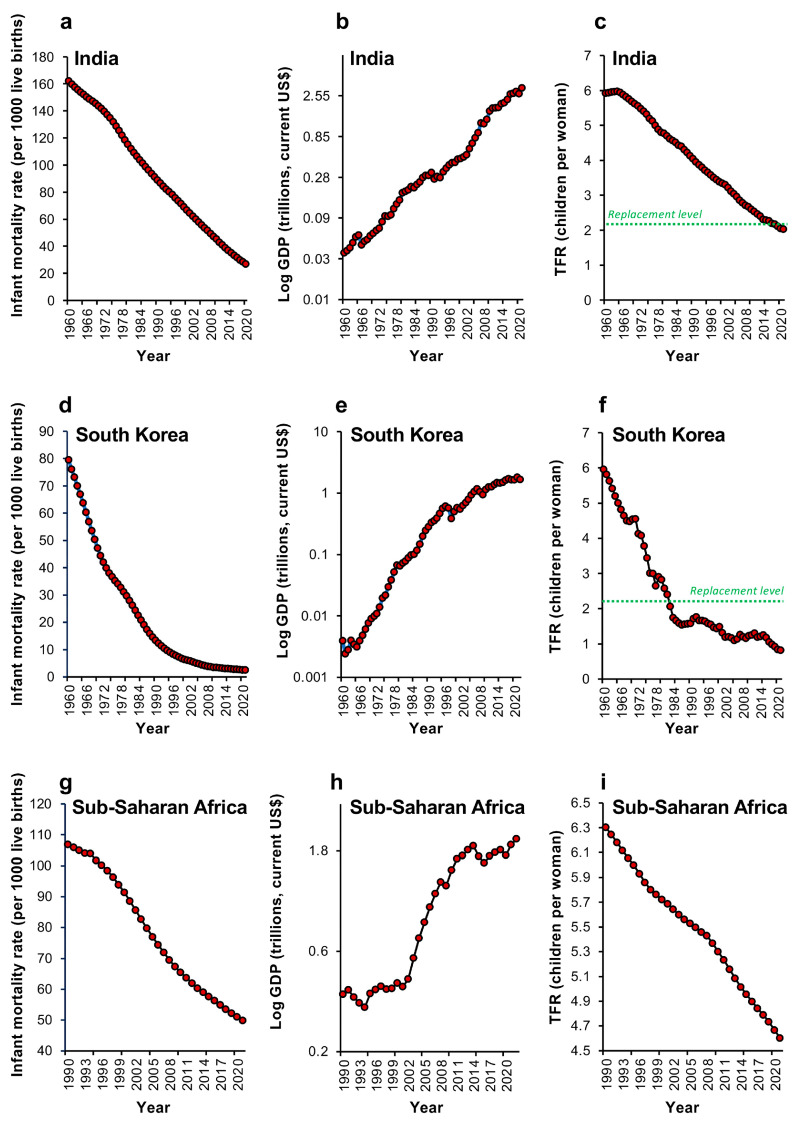
Changes in infant mortality, GDP and TFR for India, South Korea and Sub-Saharan Africa since 1960; (**a**–**c**) India; (**d**–**f**) South Korea; and (**g**–**i**) Sub-Saharan Africa. Although these countries are at very different stages of the demographic transition, they all show the same general trend with a significant decline in infant mortality being associated with a parallel fall in TFR. In India and South Korea, these changes were associated with a marked expansion of their economies, as indicted by the simultaneous increases in their respective GDP values. However, in Sub-Saharan Africa, which is at a much earlier stage of the demographic transition, the declines in infant mortality and TFR were observed prior to any significant growth in the economy, suggesting the primary importance of decreased infant mortality in triggering the fertility decline associated with the demographic transition. Each red circle represents data for a single year. Source: World Bank Open data (2023).

At a national level, the demographic transition is beautifully exemplified by India (Figure 2a–c). Beginning in 1960 we see a progressive decline in infant mortality rates associated with a sustained increase in economic prosperity (GDP). TFR increases slightly in response to the optimism engendered by a growing Indian economy but then commences the fall to sub-replacement levels of fertility, characteristic of the demographic transition (Figure 2a–c). Similarly, in a tiger economy such as South Korea, we see exactly the same decline in infant mortality over the past 62 years in association with impressive levels of economic growth (Figure 2d,e). Over the same period of time, TFR rates fell dramatically to sub-replacement levels (Figure 2f). Even in sub-Saharan Africa, for which World Bank data are available from 1990 (Figure 2g–i), infant mortality and TFR levels have also declined over the past 33 years, even though the aggregate fertility for this group of nations is still well above replacement levels. Interestingly, in the case of Sub-Saharan Africa, which is at a very early stage of the demographic transition, TFR decline was not apparently triggered by an increase in prosperity, at least as reflected in GDP, but was tightly correlated with a fall in infant mortality.

**Figure 3:**
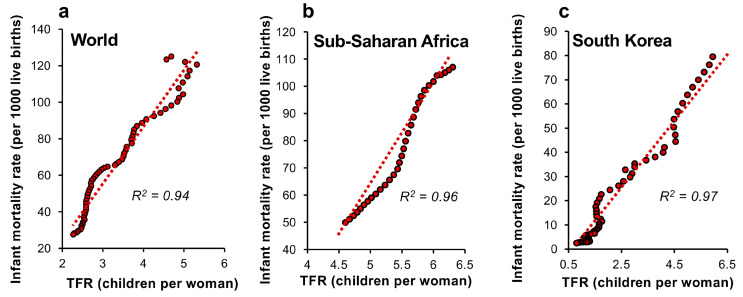
The strong relationship between decreasing infant mortality and the total fertility decline associated with the demographic transition since 1960. Linear regression analyses of the relationship between infant mortality and TFR for (**a**) the world, (**b**) Sub-Saharan Africa and (**c**) South Korea. All data sets show a high correlation coefficient (R^2^ value) linking infant mortality and TFR. Each red circle represents data for a single year. Source: World Bank Open data (2023).

Decreases in infant and child mortality rates have indeed been proposed as agents of fertility decline because they accelerate urbanization and allow the increased investment in human capital needed to drive economic growth [40]. Although the primary importance of decreased infant mortality in precipitating fertility decline has been controversial [41], the overall evidence supports a central role for this factor in the aetiology of falling TFR [42]. According to Cleland [43] ‘nowhere in the world, independent of the time period, the levels of wealth or the degree of modernisation, has fertility change taken place without significant prior mortality change’. In support of this concept, a plot of global TFR against infant mortality rates from 1960 onwards shows an extremely tight linear correlation between these criteria; the decline in infant mortality being exactly paralleled by the decline in TFR (R^2^ = 0.94; *p* < 0.001; Figure 3a). Precisely the same powerful relationship is seen countries at opposite ends of the developmental scale from Sub-Saharan Africa (R^2^ = 0.96; *p* < 0.001) to South Korea (R^2^ = 0.97; *p* < 0.001) (Figure 3b,c).

## 5. Mechanisms Responsible for TFR Decline

The social and cultural factors influencing the reproductive choices that drive the demographic transition have been reviewed many times and will not be extensively discussed here. While reductions in mortality, particularly infant mortality, and increased prosperity are regarded as the keys to initiating this process, there are many other factors that contribute to, and support, this demographic change. Decision-making around the notion of an ‘ideal family size’ [44,45] is influenced by prior family history and the offspring quantity–quality trade-off, enhancing the per capita investment in individual children at the expense of family size [46]. Low fertility is also supported by the spread of ideas concerning the nature of modern society and captured in concepts such as ‘developmental idealism’ and ‘reflexive modernization’ that, among other things, extol the virtues of low fertility, greater autonomy and the primacy of individual fulfilment [47,48,49,50,51]. An ideational change that is critical in this context is the social aspiration for gender equality and the associated mass entry of women into the labour market. This shift of female focus away from procreation towards professional fulfilment has led to a delay in childbearing, which is a particular issue for humankind because, unlike most other mammalian species, our fertility declines dramatically after the age of 38 [1,52,53,54]. Moreover, as I shall discuss later in this article, assisted reproductive technologies (ARTs) can do little to support women in this age category because the live birth rate following ART declines in exactly the same manner as we see in naturally conceived pregnancies [55]. As a result, many women in modern society have families that are smaller than they would wish [56] and there is a growing acceptance of childlessness as a positive lifestyle choice [57,58,59,60].

The entry of women into the modern workplace is also associated with the progressive urbanization of our species [40]. Urban environments are associated with the improved availability of contraception which assists women in postponing their childbearing years [61,62,63]. Urbanization also promotes a decline in fertility because it reduces the financial incentive to have a family, fuelled by the paucity of affordable housing in major cities and low levels of disposable income [62,63,64,65].

## 6. Transition to Sub-Replacement Fertility

The above range of socioeconomic and cultural factors drive down fertility rates, their demographic power being reflected in the speed at which the fertility decline occurs. In the tiger economies of Asia (Singapore, Hong Kong, Singapore and South Korea) it took an average (±SE) of just over 20 years (20.05 ± 2.02) to shift from a pre-transition TFR of >5 to a sub-replacement value of ≤2. In China, the same transition took 23 years. Even Albania, which banned all forms of contraception until 1992, managed to decrease its TFR from 6.455 in 1960 to a sub-replacement value of 2.036 in 2002; a journey that took only 42 years. World Bank Open Data (2023) lists 136 independent countries that had a TFR or more than 5 in 1960, 44 (32.3%) of which went through a demographic transition to sub-replacement levels (TFR < 2.1) in an average of 46.79 ± 1.66 years.

The point of this analysis is to emphasize that, when the conditions are right, the transition to sub-replacement TFR occurs relatively rapidly, generally being accomplished within 20–50 years. Importantly, this is too fast for the traditional machinery of biological evolution, involving the key processes of mutation, variation and natural selection to impact the reproductive profile of entire nations, even though such mechanisms might be effective in isolated communities [66]. Of course, natural selection is constantly shaping the human genotype even during the demographic transition, such that any chromosomal or gene mutation that causes a serious loss of fertility will be rapidly selected against and removed from the population; the three most common genetic causes of human infertility, Klinefelter syndrome (XXY), Turner syndrome (XO) and Y-chromosome deletions being cases in point. However, such natural selection mechanisms are designed to support fertility, not diminish it. So, the decline in TFR cannot be driven by evolutionary mechanisms in the traditional biological sense of the word; it must be a socioeconomic phenomenon driven by the factors listed above [4,67,68,69].

From a biological perspective, the changes associated with the first demographic transition, have taken place so rapidly that our fundamental reproductive genotype, and thus our basic fecundity, has had no opportunity to adjust. Prior to the demographic transition, our genetic constitution would have been fine-tuned to maximise our reproductive fitness in a world where infant mortality rates were high, and life was short. In those countries that have now transitioned to sub-replacement TFRs, we have a discordant situation where the reproductive genotype is still in ‘pre-transition, high fertility mode’ while reproductive output (fertility rate) is on a ‘post-transition, lowest-low’ setting. What are the consequences of this discrepancy? It is an important question to ask because it relates to the reversibility of the fertility changes associated with the demographic transition. If future circumstances so demand, shall we be able to adopt a pronatalist stance and increase our fertility rate once again, or are we going to become snared in an infertility trap from which any return to pre-transition fertility levels may be challenging [1]?

The post-transition phase of demographic transition, sometimes referred to as the second demographic transition, is characterized by consistently low fertility rates, the dissolution of the nuclear family, and a disconnection between marriage and procreation [53,70]. Many of the world’s most advanced economies have now reached this phase of development, particularly in Europe, North America, Japan, China, the tiger economies of Asia and Australasia. These are unchartered demographic waters. What happens when the TFR falls below the replacement threshold for prolonged periods of time?

## 7. Post-Transition Societies

### 7.1. Sub-Replacement Fertility Levels Are Difficult to Reverse

**Figure 4:**
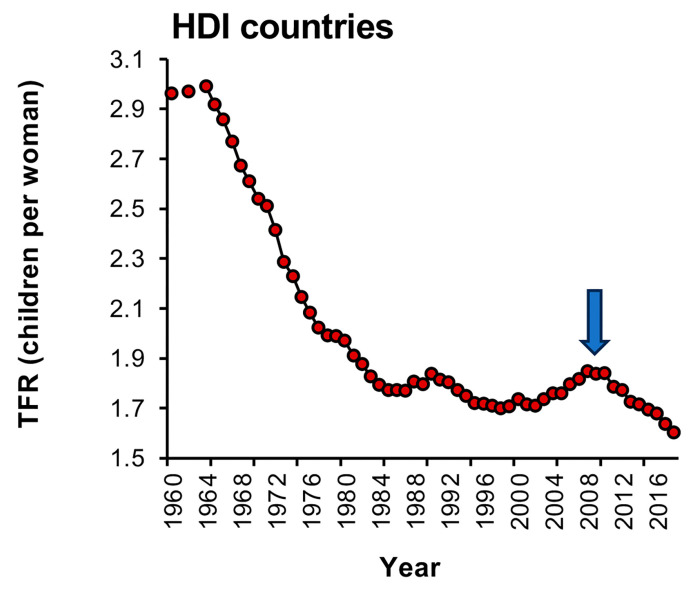
Changes in TFR once sub-replacement values have been reached. The demographic transition delivers sub-replacement levels of fertility so rapidly that the genetic underpinnings of fecundity do not have an opportunity to change. The result is that, in the early stages of the second demographic transition, fertility rates should be amenable to manipulation. In support of this concept, Myrskylä et al. [72] identified a cohort of advanced economies exhibiting high human development indices (HDIs) that exhibited an increase in fertility between 2000 and 2009 (arrowed). This increase was only transient however, and since 2010, these same countries have continued their descent into extremely low levels of fertility [1]. Each Red circle represents data for a single year. Source: World Bank Open data (2023).

One school of thought that dominated population predictions from the United Nations was that societies would eventually converge and stabilize their TFRs close to the replacement level, leading to a gently falling but stable population outlook. When this view was articulated in the World Population Prospects report of 1998 [71], it was certainly welcome news for the member states concerned about population numbers. Comforting news was also provided by Myrskylä et al. [72], who found that advanced economies, with a high human development index, are capable of a certain level of fertility recovery as a result of continued economic and social development. Thus, as societies advance and fertility rates fall, policies and strategies are introduced to restoke the fires of fertility and stabilize population growth at the appropriate level. This analysis identified 18 countries which had turned the corner in this manner including Norway, the Netherlands, the United States, Denmark, Germany, Spain, Belgium, Luxembourg, Finland, Israel, Italy, Sweden, France, Iceland, the United Kingdom, New Zealand, Greece and Ireland. If we now look at recent fertility rate data for these countries (Figure 4), it is evident that there was indeed an increase in TFR for these highly developed countries that peaked in around 2009, as reported by Myrskylä et al. [72]. Unfortunately, however, this increase was short-lived and over the past decade the mean TFRs for these countries have declined once more (Figure 4). Thus, once TFRs have descended below the replacement threshold, it appears to be very difficult to reverse the process in a sustained and meaningful way, other than by immigration [73].

### 7.2. The Cultural Forces That Keep Fertility Levels Low—The Power of One

It is now very apparent that societies that have entered the post-transition phase of demographic change and reached sub-replacement levels of fertility show no sign of stabilization or a return to replacement TFR levels [53,74]. Why would they? The socioeconomic factors that drove the decline in TFR in the first instance are still there and still putting downward pressure on the ability, or even the desire, of young couples to have children. These socioeconomic factors seem to be not only maintaining low levels of fertility in post-transition societies but creating record ‘lowest-low fertility rates’ [75,76]. Countries with a TFR below 1.5 now include many examples from Europe (Spain, Portugal, Italy, Greece, Luxembourg, Malta, Bosnia and Herzegovina and Moldova), as well as East and Southeast Asia (South Korea, Hong Kong and Singapore). There are some countries, particularly in Eastern Europe, where a low TFR in combination with other factors, including significant emigration by individuals seeking a better life elsewhere, are leading to rapid depopulation (Albania, Latvia, Lithuania, Bulgaria and Estonia) [77]. Conversely, there are other counties with low TFRs whose populations are growing entirely due to high rates of immigration (the United States, United Kingdom, Australia, Canada, United Arab Emirates and Germany) as individuals endeavour to improve their security, wealth and quality of life by moving to the more prosperous corners of the globe. Either way, migration is having a significant impact on demographic change. Thus, while a majority of the world’s population still live in their country of origin [78], migration numbers are at an all-time high, fuelled by exactly the kind of personal autonomy and individualism pioneered by the Enlightenment, three centuries ago.

We have now entered a stage of socioeconomic development when the concepts of independence, autonomy and personal freedom espoused in the 18th century by luminaries such as Rousseau and Kant are beginning to have demographic impact. Modern Western society, and the social networking tools that support it, is driven by a brand of individualism that prizes autonomy, security and self-fulfilment above all else. It encourages behaviours such as migration to achieve these ends and prioritizes personal wellbeing above procreation or devotion to a particular deity, as representing the meaning of life. Once achieved, personal liberty and autonomy are not easily surrendered. As a result, the primacy of the individual and the globalization of individualist values will be with us for the foreseeable future [79] and are likely to mean that our descent into sub-replacement fertility territory will be difficult to reverse from a cultural standpoint. Indeed, the spread of Westernized culture is such that it is tempting to commit the cardinal sin of ‘reading history sideways’ [49] and suggest that all countries are on the same cultural journey, with the same demographic destination—a protracted stay in lowest-low fertility land. Should this prediction materialize, and all advanced economies experience prolonged periods of extremely low fertility, what are the biological consequences? In particular, will societies experiencing sub-replacement levels of fertility for prolonged periods of time experience any lasting damage to their fundamental fecundity and thus their potential reproductive fitness?

## 8. Long-Term Consequences of Sub-Replacement TFR

The low fertility and low infant mortality that characterize post-transition countries mean that the evolutionary selection pressure that originally refined and calibrated the reproductive genotype of our species, has been lost. In the absence of such pressure, the reproductive potential of individual couples is not being tested, and high-fertility genotypes have limited advantage over their low-fertility counterparts. In modern society, with vanishingly low levels of infant mortality, a couple could possess a combined high-fertility genotype capable of delivering 15 children and decide they want to remain childless in order to pursue their professional careers. Alternatively, a couple may possess a combined sub-fertility genotype incapable of generating any children at all, and yet elect to have three children by ART. In modern society, selection for high-fertility genotypes has been obliterated. Of course, natural selection has not stopped in post-transition societies and mutations that cause sterility, such as Y-chromosome deletions, will still be weeded out very rapidly. However, genes that cause subfertility will not be strongly selected against in a society with an average TFR of 1.5 or less. As a result, we would expect such poor-fertility genotypes to accumulate within the population and to increase the level of overall variance in genes controlling reproduction. There is already emerging evidence for an increase in such variance in populations that are experiencing sub-replacement levels of fertility [80]. As advanced methods of large-scale genetic sequencing become available, so our capacity to address this question at a whole-genome level will improve. We already know that genetic causes of human infertility are significant and may account for up to 40–50% of all cases [81]. Furthermore, new mutations are being unearthed on a regular basis as our capacity to screen for pathogenic genetic variants in the patient population increases [82,83,84]. The relaxation of selection pressure on high-fecundity genotypes during the post-transition era inevitably means that the incidence of such variants in the population will increase with the passage of time and, since most variants are neutral to deleterious, our fecundity will suffer.

In addition to the impact of genetic factors on the reproductive potential of our species, we should also not forget that we increasingly live in highly polluted urban environments, featuring industrial chemicals that have a known capacity to suppress fertility and induce reproductive cancers [85,86,87]. These reproductive toxicants will clearly contribute directly to a post-transition decline in fecundity as a result of their capacity to interfere with multiple stages of the reproductive process. They may also have an indirect impact on human reproduction through their ability to increase DNA damage in the male germ line and thereby increase the incidence of mutations carried by the offspring, some of which may both impact fertility and the incidence of cancers typical of modern, industrialized societies [88,89].

Not all of the evolutionary news may be bad, however. A great deal of human infertility is due to delayed childbearing, with women initiating their families so late that they do not achieve their desired family size before the portcullis of age-dependent infertility descends [90]. Under these circumstances we may see active selection for genotypes that support reproduction later in life. In advanced economies, the age at which women have their first child is clearly increasing [91] and it will be interesting to see if this trend ultimately selects for genotypes commensurate with the retention of fertility beyond the age of 40.

## 9. Role of the Assisted-Conception Industry

Any tendency towards low fertility in the post-transition era will potentially be exacerbated by the assisted conception industry that, as a by-product of its success, automatically retains poor-fertility genotypes within the population. Up to this point, the IVF industry has not operated at a scale where impacts on overall population dynamics would have been a concern. However, given the increasing uptake of such therapy by young couples seeking to start a family and the clinical tendency to treat every case of infertility with ART regardless of etiology, this day may soon arrive. In some countries, such as Denmark and Australia, more than 5% of newborns are currently conceived by ART, and this trend is inexorably moving upwards [1,2]. If this tendency continues unabated, then the assisted conception industry may make a substantial contribution to the very pathology it is trying to address. Inevitably, the more we use assisted conception in one generation, the more we are going to need it in the next. This is particularly the case with technologies such as ICSI (intra-cytoplasmic sperm injection), which compensates for failures of natural conception by physically injecting the spermatozoon in the egg.

The value of ICSI in combating severe male-factor infertility has led to a dramatic expansion in its use [92,93]. In addition, this insemination procedure is now commonly used as a default treatment for all kinds of infertility and, as a result, it is now the most widely used ART in infertility centres [93]. The widespread application of such a highly invasive IVF procedure will ensure that the poor-fertility genotypes that nature would have eliminated from the population will be artificially retained. This is undeniably the case with AZFc mutations on the Y chromosome which, because this chromosome lives alone and is passed directly from father to son, can not only cause severe infertility in men but, following ICSI, their male offspring [94,95]. This will also be the case with other genetic causes of male infertility, such as multiple morphological abnormalities of the sperm flagellum which are genetically determined but treatable with ICSI [96]. As well as facilitating the vertical transmission of infertility-inducing mutations, ICSI may also result in the creation of de novo mutations in the offspring due to the aberrant repair of sperm DNA damage in the newly formed zygote [88,97]. Such de novo mutations may then result in compromised fertility in the offspring affecting oocyte maturation, embryo development and normal testicular function [98,99,100,101].

So, as our reliance on ART to procreate increases, so the fecundity of the very population this technology is designed to help, will decline. This is not to be critical of the IVF industry, which is doing everything it can to make ART a safe and effective clinical intervention. There is no immediate threat to our reproductive fitness from the ART industry and it may take generations for the genetic changes induced by ART to become manifest at a demographic level. Nevertheless, it should be recognised that the longer we remain in low-fertility environments typical of the second demographic transition, and the more we resort to ART to compensate for declining fertility, the poorer our genetic reproductive fitness will become. With the passage of time, fertility and fecundity, which were both high pre-transition, may become realigned in post-transition societies to establish a new low-fertility equilibrium.

## 10. Is Fecundity Changing?

To determine whether the above hypothesis is correct, we shall have to monitor human fecundity as a matter of priority. Discriminating fertility and fecundity in post-transition societies will be extremely difficult because there are so many political, social and cultural overlays to reproductive health that our underlying fecundity is often obscured from view [102]. Data on the rapid secular decline in sperm counts is certainly suggestive of an environmental impact on human fecundity. Although the validity of these data has been repeatedly questioned [103,104,105], recent analyses have not only confirmed the reality of this change but also emphasized its global nature. Declining sperm counts have now been recorded in South America, China and Africa, as well as Northern Europe, North America and Australasia [106,107]. In parallel with the decline in semen quality, as populations have engaged the second demographic transition and fertility rates have descended to sub-replacement levels, so the incidence of testicular cancer has risen exponentially [1,2]. Such information, as well as a wealth of animal data, reinforce the notion that advanced modern societies expressing sub-replacement levels of fertility are exposed to environmental contaminants as well as other lifestyle factors, such as diet and obesity, that are making an indelible mark on our reproductive health [86,108]. However, whether the decline in sperm counts has yet amounted to a change in fecundity is open to question [109]. The traditional descriptive criteria of semen quality, like sperm count, have a very limited correlation with the overall fecundity of patients in prospective studies [110]. Furthermore, even though human sperm counts may have declined, they are, even in the most adversely affected nations, still with the normal range [106]. The most concerning aspect of the sperm count narrative is not the absolute number of spermatozoa in the ejaculate but that the direction of change is showing no sign of stabilization or reversal; indeed, the pace of decline actually seems to be accelerating [106]. So, even though there is no evidence that the fall in sperm counts is affecting fecundity at present, we cannot rule out the possibility that this may be the case sometime in the future [111].

Importantly, the male is not alone is his vulnerability to toxicant exposure. Female reproduction may also be vulnerable to attack by environmental toxins, precipitating a range of conditions including polycystic ovary syndrome, primary ovarian insufficiency, multi-oocytic follicles and meiotic defects including aneuploidies [112]. Thus, there appear to be significant threats to reproductive health in modern environments that cut across gender boundaries. However, even if environmental toxicants are responsible for major changes to our reproductive health [113], questions still remain as to the heritability of such changes. This is a critical point because, if a permanent genotypic change is involved, fecundity might not be readily restored once the offending pollutants have been removed from the environment.

There can be no doubt that many of the toxicants characterizing modern environments are powerful mutagens—either by direct action or through the induction of oxidative DNA damage. Such widespread reproductive toxicants as bisphenols and phthalate esters are known to induce oxidative DNA damage in both the male and female germ line [114,115,116,117,118] and are acknowledged mutagens [119,120]. These compounds also induce multigenerational infertility in animal models [121,122,123], strongly suggesting they are capable of leaving an indelible mark of the future fertility of our species through a combination of genetic and epigenetic effects [124,125].

Experimental studies have also indicated that tobacco smoke can cause mutations in the male germ line, impacting the mutational load carried by the offspring and inducing transgenerational pathological change [126,127]. Such damage is thought to underpin the increased incidence of childhood cancer, particularly leukemias, in the offspring of males who smoke heavily [128,129]. In addition, strong data exist indicating that both maternal and paternal smoking can have a significant impact on semen quality in male offspring [130,131]. In combination, these data suggest that exposure to environmental toxicants can have a transgenerational impact on fecundity via genetic/epigenetic pathways [132,133], and may contribute to the establishment of sustained levels of low fertility in polluted, post-transition societies.

Another potential indicator of a change in human fecundity, as societies experience low fertility, is a progressive increase in the uptake of assisted-conception therapy [1,2,134]. The major problem with ART uptake as a measure of human fecundity is that it is subject to distortions due to factors such as the extent to which public funding is available to resource this therapeutic procedure, the severity of the regulatory frameworks governing such treatment, and international differences in sociocultural norms pertaining to reproductive behaviour. In some countries, governmental support for assisted conception is very good (e.g., Scandinavia or Australia), whereas in others it is quite poor (e.g., USA) [135]. The increase in assisted-conception uptake is indicative of a decline in human fecundity; however, these criteria are too severely influenced by external forces to provide any measure of proof that such a change is occurring.

Fecundity may be more directly assessed by determining the incidence of infertility (failure to achieve a pregnancy after 12 months or 5 years of unprotected intercourse) or the time-to pregnancy (TTP). The specific methodological approaches used to assess human fecundity are still not fully developed and currently lack consensus [136,137]. As a result of this lack of standardization, there are currently no data available on whether the fecundity of our species is changing with the passage of time, particularly in countries that are experiencing extremely low fertility levels. As emphasized by te Velde et al. [15] more than a decade ago, we still do not have a validated surveillance system for monitoring this reproductive criterion. Whether human fecundity is declining is clearly an answerable question that needs to be addressed as a matter of urgency if we are to manage our reproductive health effectively. Developing the appropriate instruments for monitoring the long-term fecundity of human populations experiencing prolonged periods of low fertility should, therefore, be a priority for the future.

## 11. Conclusions

**Figure 5:**
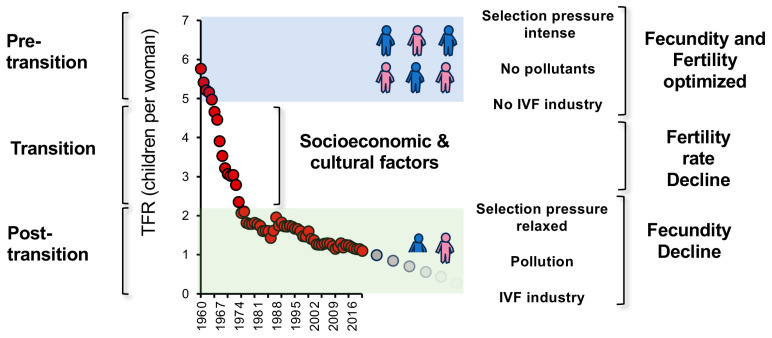
Changes in fertility and fecundity during the demographic transition. During the pre-transition period of human development, fecundity was calibrated to deliver a fertility rate of 5–7 children per woman during her reproductive lifespan. Once instigated, the demographic transition is capable of delivering a very rapid decline in fertility rates to sub-replacement levels (<2.1 children per woman) within 1–2 generations. This decline in fertility is induced by a range of cultural and socioeconomic factors and occurs so rapidly that the genetic basis of fecundity remains essentially unchanged. As a result, we enter the second demographic transition with low levels of fertility but sufficient fundamental fecundity to reverse the fertility decline with appropriate pro-natalist policies, should we so wish. However, the longer societies remain in this post-transitional state, the more fecundity will come under threat as a consequence of the presence of reproductive toxins in the environment, the relaxed evolutionary selection pressure on high-fecundity genotypes and the ability of the assisted-conception industry to keep poor-fertility genes in the population when operated at scale. If these factors collude to seriously impact human fecundity, we risk falling into an infertility trap that will permanently impact the reproductive capacity of our species [1]. Source: World Bank Open data (2023).

A dramatic fall in fertility rates appears to be a universal phenomenon that affects all societies as they engage the demographic transition. Although much has been written about the socioeconomic aspects of this transition in terms of proximal causation and future consequences, little consideration has been given to this process from a biological perspective. For much of human history, our species has been characterized by fecundity settings that have allowed women to have 5–7 children over a lifetime, which more or less balanced the high rates of infant mortality that blighted the earliest stages of human evolution and kept population growth in check (Figure 5). Following the global wave of prosperity and awareness initiated by the Industrial Revolution and the Enlightenment in 18th century Europe, societies have experienced a demographic transition and the significant fall in fertility rates that accompanies this process. This decline has been relatively rapid, resulting in a fall to sub-replacement fertility rates within 1–2 generations, and was largely achieved on the basis of socioeconomic and cultural factors, including the entry of educated woman into the modern workplace and the associated delay in childbearing, urbanization, the increased availability of contraception and a reduction in our fundamental motivation to procreate.

At the end of this phase of the demographic transition, societies enter the post-transition phase in a reproductively discordant state: total fertility rates are extremely low, while the genetic underpinnings of fecundity will still be at pre-transition levels, given the speed at which the transition occurs (Figure 5). The retention of high-fecundity genotypes means that, in the short-term, societies will still have the flexibility to return to high levels of fertility if they so desire. However, with the passage of time, this flexibility may erode due to the relaxation of selection pressure on high fecundity genotypes, aided and abetted by the genetic damage introduced by environmental pollution and an assisted conception industry that, when operated at scale, will ensure that subfertility genotypes are retained within the population. The result will be a gradual fall in fecundity that will then enhance the low fertility rates found in post-transition societies in a self-promoting cycle (Figure 5). These changes will take time. Population momentum will ensure that even though fertility rates are falling, the global population will continue to expand until we reach the zenith of the population curve at around 10.4 billion in 2080 or thereabouts. However, once this momentum has become exhausted, we shall see a period of rapid population decline that will impact some societies earlier than others. The populations of countries like Japan, South Korea, China and Bulgaria are already in retreat and will generate economic, social and geopolitical consequences in the next 2–3 decades. However, the forces driving down human fertility are so pervasive and ubiquitous that all countries will ultimately experience the same fate, even those in Sub-Saharan Africa. This is not necessarily a bad thing. Declining population numbers should have positive global impacts on climate change, the spread of pandemics, levels of environmental pollution and the preservation of biodiversity. However, at a national level, rapid depopulation will also present challenges in terms economic prosperity, the provision of infrastructure and the management of political power. If we are to engineer a soft landing, gaining some measure of control over population dynamics will be critical and achievable, providing we are aware of the mechanisms at play and can prevent the closing of an infertility trap that will shape the long-term destiny of our species.

In the short term, the policies and strategies that governments should introduce to reverse this low-fertility trend should be designed to encourage young couples to have children earlier in life than is currently the case. These measures might include taxation reform to substantially shift this burden away from those aspiring to have a family. Encouragement to have children could also come in the form of more affordable housing, increases in the availability of childcare, generous parental leave schemes, baby bonuses and revised superannuation schemes that do not penalize couples for the time spent in having and rearing a family [1,138]. We might also adopt policies to encourage retirees back into work, seek ways of improving the efficiency of the aged care system and carefully adjust the immigration rheostat to provide short-term compensation for our reproductive shortfalls [1,139]. In the longer term, effective monitoring of our environment for reproductive toxicants and decreased reliance on ART as a default treatment for human infertility might also be helpful [1]. Research into the productive use of AI and development of high levels of automation to compensate for skilled-labour shortages, should also help maintain productivity in the face of falling population numbers. It would also help if society realized that one of the qualities we cannot change about the world we live in is our fundamental biology. If employers are going to reap the rewards of having women in their workforce, they also need to accommodate the realities of reproductive life and support their employees in their quest to find a balance between family life, professional advancement and a well-supported retirement [1]. A full analysis of the approaches we might adopt to control, rather than fall victim to, future changes in population dynamics is beyond the reach of this particular review. However, we should be able to agree that change is inevitable and that our aim should be to provide a combination technologies and policy settings that will enable young couples to achieve their desired family size—whatever that might be.
